# HIF-1α downregulation and apoptosis in hypoxic prostate tumor cells infected with oncolytic Mammalian Orthoreovirus

**DOI:** 10.18632/oncotarget.1767

**Published:** 2014-01-31

**Authors:** Pooja Gupta-Saraf, Cathy L. Miller

**Affiliations:** ^1^ Department of Veterinary Microbiology and Preventive Medicine, Iowa State University, Ames, IA; ^2^ Interdepartmental Genetics Program, Iowa State University, Ames, IA; ^3^ College of Veterinary Medicine, Iowa State University, Ames, IA

**Keywords:** HIF-1α, hypoxia, prostate cancer, Reovirus, viral oncolysis

## Abstract

Hypoxia has emerged as one of the most important drivers of tumor aggression, metastasis, and poor clinical outcome in many cancers. In prostate cancer (PCa), hypoxia has been strongly correlated to biochemical failure and local recurrence. However, current PCa treatment options do not address hypoxic cells highlighting a critical gap in existing therapies and the need for development of therapies that target hypoxic prostate tumor cells. Mammalian orthoreovirus (MRV) is an oncolytic virus that targets tumor cells over normal cells which has been shown to be safe and effective against many cancers *in vitro*, in animal models, and in human clinical trials. We found that MRV infects and replicates in hypoxic prostate tumor cells to levels comparable to normoxic cells leading to apoptosis and cell death. In addition, the regulatory subunit (HIF-1a) of the master transcriptional regulator of hypoxia, HIF-1, was significantly downregulated in infected cells. HIF-1a downregulation was found to occur via ubiquitin-dependent proteasome-mediated degradation and translational inhibition. Virus-mediated HIF-1a degradation required the HIF-1a PAS domain and expression of the receptor for activated kinase C (RACK1) protein. These data provide evidence that MRV may be a viable therapeutic option for targeting hypoxic cells and HIF-la in PCa.

## INTRODUCTION

Disease progression and mortality in cancer patients is often correlated with the presence of chronic or transient hypoxic microregions within tumors [1–3]. Hypoxia induced changes can lead to tumor propagation through the selection of hypoxia-adapted mutant cells. Cells develop hypoxia adaptation through the upregulation of genes encoding proteins that promote proliferation, apoptosis resistance, and angiogenesis [4, 5]. Upregulation of these genes in turn leads to increased tumor invasion and metastasis, resulting in disease progression and poor clinical outcome [6, 7]. Moreover, hypoxic tumor cells are often resistant to chemotherapy and radiotherapy, posing further challenges to the development of treatments that will lead to a complete cancer cure [8, 9].

Prostate cancer (PCa) is the second leading cause of cancer death in men in the United States [10]. Therapies, in the form of surgical removal and radiotherapy, have undesired side effects such as impotency and urinary incontinence [11]. Hormone therapy, consisting of surgical or chemical deprivation of androgen, is an additional treatment option for PCa patients. However, most prostate tumors develop androgen resistance within one to three years of therapy onset and resume growth. Detection of molecular markers of hypoxia in prostate tumors has been linked to progression, poor prognosis, low survival rate and early biochemical relapse [12, 13]. Considering that prostate tumors extract benefit from poor oxygenation for survival and metastasis, there is a crucial need for the development of therapeutics targeting hypoxic PCa cells.

The upregulation of proteins involved in adaptation and survival in the hypoxic environment is controlled by the transcription factor, hypoxia inducible factor 1 (HIF-1). HIF-1 is a heterodimer of two helix-loop-helix Per-ARNT-Sim proteins, HIF-la and HIF-1β. When HIF-1α and HIF-1β are present, they form a dimer, translocate to the nucleus, and bind to canonical DNA sequences termed hypoxia response elements (HREs) in the promoter or enhancer regions of target genes [14]. The formation of HIF-1 is dependent on the availability of HIF-1a, which is tightly regulated post-translationally via oxygen-dependent and -independent degradation pathways. Under normoxic conditions, HIF-la is hydroxylated by prolyl hydroxylase 2 (PHD2) at proline residues 402 and 564 [15, 16]. This leads to binding of von Hippel-Lindau (pVHL) protein to the HIF-la oxygen dependent degradation (ODD) domain. pVHL recruits an E3 ubiquitin-protein ligase complex to HIF-1α, resulting in ubiquitination and degradation of HIF-1α by the 26S proteasome [17–19]. Under hypoxic conditions the action of PHD2 is inhibited and HIF-1α is stabilized. An oxygen-independent pathway for HIF-la degradation is regulated by the receptor for activated C kinase (RACK1) protein, which competes with heat shock protein 90 (HSP90) for binding to the HIF-1α PAS domain [20]. Binding of HSP90 leads to stabilization of HIF-1α and binding of RACK1 to HIF-1α leads to its ubiquitination and proteasome mediated degradation by an E3 ubiquitin ligase complex [20].

Mammalian orthoreovirus (MRV) is a clinically benign, double stranded RNA family *Reoviridae* member that is not associated with major pathogenicity in humans or other animals. MRV is an oncolytic virus that preferentially replicates in tumor cells over normal cells [21]. Successful demonstration of MRV oncolytic activity against numerous cancer types in animal models [22–24] led to its development and clinical testing as a cancer therapy in a number of Phase I/II/III human clinical trials. It has emerged as a safe and effective therapy for a number of cancer types leading to disease stabilization and tumor regression in many patients [25–28]. With regards to PCa, MRV specifically replicates in and kills PCa cells *in vitro*, in an *in vivo* animal model, and in human patients [29, 30].

Little is known about the impact of MRV infection on hypoxic tumor cells. One previous study suggested that MRV infection leads to decreased levels of HIF-1α in hypoxic lung, colon, and renal tumor cells in a manner dependent on proteasome inhibition but independent of VHL expression. This study also suggested that MRV protein expression was inhibited in VHL-/- cells that constitutively express HIF-la and that MRV infection induced cell death through the activation of caspase 8 and apoptosis [31]. In contrast, another study showed that MRV infection stabilized HIF-1α and induced apoptosis in a caspase independent mechanism in human glioblastoma cell lines [32]. These studies clearly indicate that the effects of hypoxia on MRV infection and the effects of MRV on the hypoxic response and cell death in tumor cells growing under hypoxic conditions is cell type specific and thus findings from one tumor type cannot be applied to another. A comprehensive investigation of MRV replication under hypoxic conditions and the mechanisms involved in MRV-induced HIF-1α regulation was not done in either of these prior studies. The objectives for this study were to examine MRV replication, impact on the hypoxic response, and induction of tumor cell death of three prostate tumor cell lines that vary in both androgen sensitivity and metastatic potential grown under normoxic and hypoxic conditions. We found that MRV readily replicates in and kills prostate tumor cells growing in hypoxic conditions, and further, that HIF-la protein levels and activity are downregulated in MRV infected prostate tumor cells. We additionally provide evidence that in PCa cells, MRV-induced HIF-la degradation requires the HIF-1α PAS domain, and is inhibited following siRNA knockdown of RACK1, suggesting that MRV induces HIF-1α degradation through the RACK1 pathway. These findings represent an important step in the characterization of MRV oncolytic treatment as a therapy for killing hypoxia adapted prostate tumor cells.

## RESULTS

### MRV is translationally active and replicates in hypoxic tumor cells

**Figure 1:**
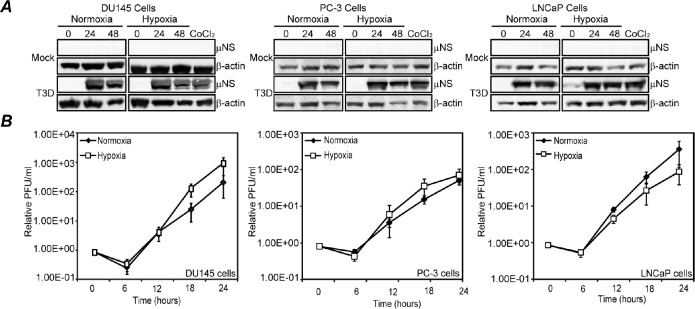
Hypoxia does not interfere with MRV translation or replication in prostate tumor cells Normoxic or hypoxic DU145, PC-3 and LNCaP prostate tumor cells were mock-infected or infected with MRV T3D and further incubated under normoxic or hypoxic conditions. CoCl_2_ was added to cells where indicated at 4 h prior to infection. A) At indicated times, cells were harvested and proteins were separated on SDS-PAGE and transferred to nitrocellulose. Blots were immunostained with rabbit α-μNS polyclonal antiserum or rabbit β-actin polyclonal antibodies followed by AP-conjugated goat α-rabbit IgG secondary antibodies. B) At 0, 6, 12, 18, and 24 h p.i., cells were harvested and subjected to three freeze/thaw cycles. Standard virus plaque assays on L929 cells were performed on cell lysates to determine virus titer (PFU). Mean of the relative PFU/ml from three independent experiments is plotted and the error bars represent the standard deviation (SD).

Hypoxia-induced shutoff of protein synthesis negatively impacts the ability of some oncolytic viruses to replicate in tumor cells growing in hypoxic environments [33, 34]. Because MRV mRNAs escape host translational shutoff induced by infection, [35], we hypothesized that MRV may replicate in prostate tumor cells growing in a hypoxic environment. To test this hypothesis, we examined MRV infection in normoxic and hypoxic prostate tumor cell lines. We examined three cell lines that represent androgen-resistant, moderate metastatic (DU 145), androgen-resistant, high metastatic (PC-3), and androgen-sensitive, low metastatic (LNCaP) prostate tumor cells. Each cell line was incubated in normoxic or hypoxic conditions for 4 h, at which time induction of the hypoxic response was evident by strong upregulation of HIF-1α (data not shown). In addition to growth in hypoxic conditions, independent samples were treated with cobalt chloride (CoCl_2_), which mimics hypoxia in the cell by inhibiting PHD2 hydroxy lation of HIF-1α [36]. Normoxic, hypoxic, or CoCl_2_-treated cells were then mock infected or infected with MRV and incubated for an additional 24 or 48 h under normoxic or hypoxic conditions. Following incubation, cells were harvested in protein loading buffer, and immunoblots against virus non-structural protein μNS were performed to examine MRV protein synthesis. We observed that virus protein translation was not substantially changed by growth under hypoxic relative to normoxic conditions, suggesting the virus was able to enter and initiate infection in hypoxic prostate tumor cells (Fig. 1A). To examine the impact of hypoxia on viral replication, we infected each cell line with MRV under normoxic or hypoxic conditions. Samples were harvested at 6 h intervals and plaque assays were performed to measure virus titers. We found that virus growth was not significantly different in hypoxic relative to normoxic conditions in any of the tested cell lines (Fig. 1B). Thus we established that MRV productively enters, translates protein, and replicates in diverse hypoxic prostate tumor cell lines in a manner indistinguishable from that measured in normoxic tumor cells. These data suggest that hypoxic conditions do not interfere with successful MRV infection in prostate tumor cells.

### MRV infection results in diminished HIF-1α protein levels and activity in hypoxic prostate tumor cells

**Figure 2:**
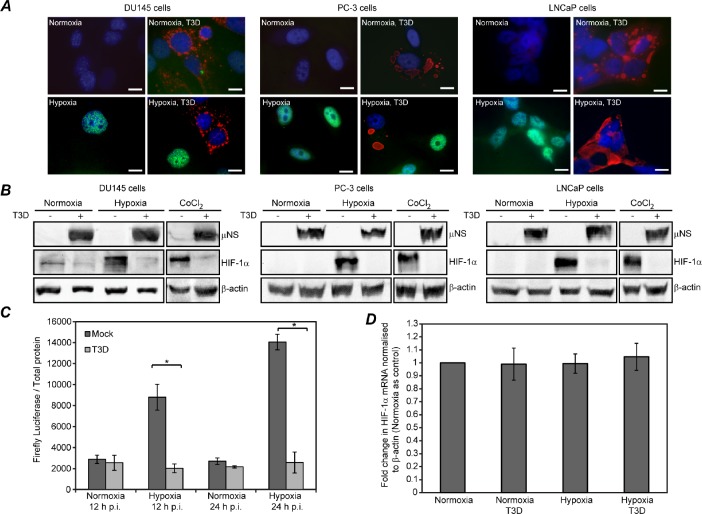
MRV infected hypoxic prostate tumor cells contain diminished HIF-lα protein levels A) Normoxic or hypoxic DU145, PC3, and LNCaP cells were mock-infected or infected with MRV T3D and further incubated under normoxic or hypoxic conditions. 24 h p.i., cells were fixed, permeabilized, and immunostained with rabbit uNS antiserum (red) and mouse HIF-la antibodies (green) followed by donkey a-rabbit Alexa 594- and donkey a-mouse Alexa 488-conjugated secondary antibodies. A merged image with DAPI is shown. Bar, 10 urn. B) Normoxic or hypoxic DU145, PC3 and LNCaP cells were mock-infected or infected with MRV T3D and further incubated under normoxic or hypoxic conditions. 4 h prior to infection, CoCl_2_ was added to cells where indicated. At 24 h p.i., cells were harvested and proteins were separated on SDS-PAGE and transferred to nitrocellulose. Blots were immunostained with rabbit a-uNS antiserum, mouse a-HIF-1α antibodies, or rabbit a-β-actin antibodies followed by AP-conjugated goat a-rabbit or mouse secondary antibodies . C) DU145 cells were transfected with pHRE-Luc and incubated under normoxic or hypoxic conditions. 24 h post-transfection, cells were mock-infected or infected with MRV T3D and further incubated in normoxic or hypoxic conditions. At 12 or 24 h p.i., luciferase activity and total protein were measured. Relative amounts of luciferase per total protein are shown. Error bars represent SD of three independent experiments. Statistically significant differences (p<0.05) are marked with ‘*’. D) DU145 cells were mock-infected or infected with MRV T3D and incubated under normoxic or hypoxic conditions. 24 h p.i., total RNA was isolated and measured using qPCR. Data from three independent experiments are represented as mean ± standard error of the mean (SEM).

In separate prior studies, MRV infection was found to induce an increase in HIF-la proteasome-mediated degradation in lung-, renal-, and colon-derived cancer cells (Cho et al, 2010), but to induce a decrease in HIF-1α degradation in brain-derived tumor cells [32], suggesting MRV infection has variable effects on the cellular hypoxic response depending on tumor type. To determine if HIF-la expression was altered by MRV infection in hypoxic prostate tumor cells, we first performed immunofluorescence assays to examine HIF-1α expression on an individual cell level. Normoxic or hypoxic DU145, PC-3, and LnCaP cells were mock infected or infected with MRV and further incubated in normoxic or hypoxic conditions. At 24 h p.i., cells were fixed and stained with antibodies against HIF-la and the MRV non-structural protein μNS. In agreement with our data suggesting hypoxia does not negatively impact MRV replication (Fig. 1), viral factories, which are key players in MRV replication and assembly, were not qualitatively different in size or number in infected cells grown in hypoxic and normoxic conditions (Fig. 2A). However, while uninfected hypoxic cells showed an increase in nuclear HIF-1α staining relative to normoxic cells, infected hypoxic cells did not show a similar increase in HIF-1α staining in response to hypoxic incubation, suggesting MRV infection may prevent accumulation of HIF-1α protein under these conditions (Fig. 2A). We next performed immunoblot assays against HIF-la in normoxic, hypoxic, or CoCl_2_-treated mock or MRV infected DU145, PC-3, and LNCaP cells. As expected, there was no accumulation of HIF-1α in mock- or MRV-infected samples grown in normoxic conditions (Fig. 2B, lanes 1 and 2 in each cell line), but abundant HIF-la protein in hypoxic and CoCl_2_ treated mock-infected samples (Fig. 2B, lanes 3 and 5 in each cell line). Strikingly, HIF-1α protein levels did not accumulate in MRV-infected hypoxic or CoCl_2_ treated cells (Fig. 2B, lanes 4 and 6 in each cell line) further suggesting MRV infection results in a strong downregulation of HIF-la protein accumulation. To extend these findings, we examined whether HIF-la protein activity was similarly diminished by MRV infection by measuring transcription from a HIF-1α-dependent firefly luciferase plasmid (pHRE-Luc) transfected into mock- and MRV-infected DU145 cells grown in normoxic and hypoxic conditions. These assays showed an expected increase in HIF-1α activity as measured by luciferase expression in uninfected hypoxic relative to normoxic cells. However, HIF-1α activity was strongly diminished in MRV-infected compared to mock-infected hypoxic cells (Fig. 2C), further supporting the conclusion that MRV infection results in diminished levels of active HIF-1α protein in hypoxic prostate tumor cells. To rule out the possibility that MRV infection was interfering with HIF-1α mRNA levels, we performed quantitative real time PCR on RNA isolated from mock- and MRV-infected DU 145 cells grown in normoxic and hypoxic environments. As shown in Fig. 2D, there was no significant difference in HIF-1α mRNA levels in these samples. Altogether, these data strongly demonstrate that MRV infection interferes with accumulation of active HIF-1α protein in hypoxic prostate tumor cells.

### MRV induced downregulation of HIF-la occurs via ubiquitin-dependent proteasome-mediated degradation and translational inhibition

**Figure 3:**
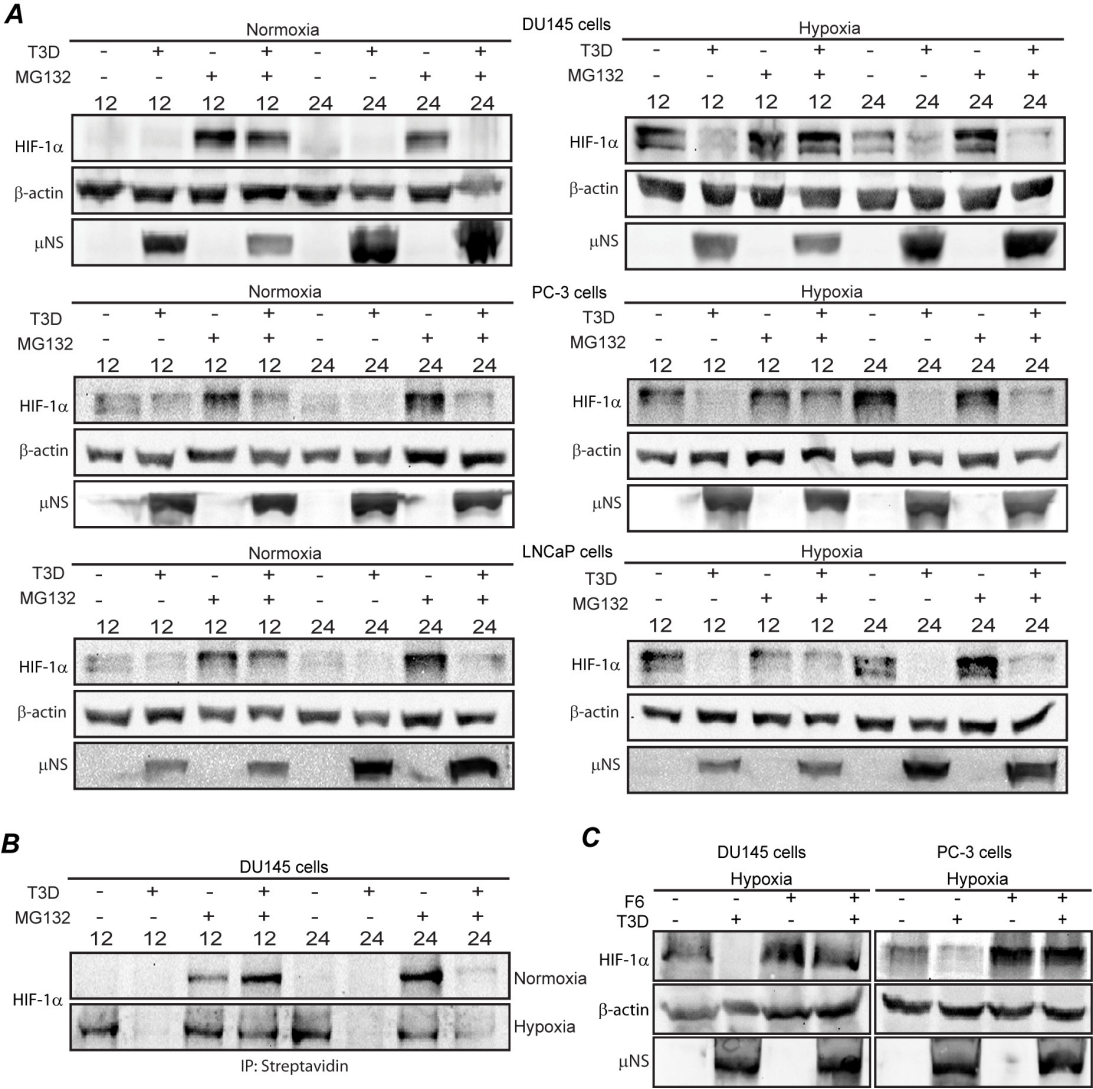
MRV induced downregulation of HIF-1α occurs via ubiquitin-dependent proteasome-mediated degradation and translational inhibition A) Normoxic or hypoxic DU145, PC3 and LNCaP cells were mock-infected or infected with MRV T3D and further incubated under normoxic or hypoxic conditions. MG132 was added to cells at 8 or 20 h p.i. At 4 h following MG132 addition, cells were harvested and proteins were separated on SDS-PAGE and transferred to nitrocellulose. Blots were immunostained with rabbit a-uNS antiserum, mouse a-HIF-1α antibodies, or rabbit a-β-actin antibodies followed by AP-conjugated goat a-rabbit or mouse secondary antibodies. B) Normoxic or hypoxic DU145 cells were mock-infected or infected with MRV T3D and further incubated under normoxic or hypoxic conditions. L-AHA was added to the cells in methionine deficient media at 6 and 18 h p.i. Cells were harvested at 12 and 24 h p.i., and conjugated to biotin-alkyne via a Click-It reaction. Proteins were precipitated using streptavidin coated magnetic beads, separated on SDS-PAGE, transferred to nitrocellulose and immunostained with mouse a-HIF-1α antibodies followed by AP-conjugated goat a-mouse secondary antibodies. C) Normoxic or hypoxic DU145 and PC3 cells were mock-infected or infected with MRV T3D and further incubated under normoxic or hypoxic conditions. At 6 h p.i. F6 was added and cells were incubated an additional 6 h, at which point cells were harvested and proteins separated on SDS-PAGE and transferred to nitrocellulose. Blots were immunostained with mouse a-HIF-1α antibodies, rabbit a-β-actin antibodies and rabbit a-uNS antiserum followed by AP-conjugated goat a-rabbit or mouse secondary antibodies

In uninfected cells under normoxic conditions, HIF-1α protein is rapidly targeted to the proteasome for degradation [19]. Thus we hypothesized that MRV induced HIF-1α downregulation in prostate tumor cells may require a functional proteasome. To test this, we examined HIF-la protein accumulation in the absence and presence of proteasome inhibitor, MG132. Mock and MRV infected DU145, PC-3, and LnCaP cells were grown in normoxic or hypoxic conditions and treated with MG132 for 4 h at either 8 or 20 h p.i., at which point cells were harvested and HIF-1α levels were measured by immunoblot. MRV infection again resulted in decreased HIF-1α levels in hypoxic tumor cells in the absence of MG132 at both times p.i. in all cell lines (Fig 3A). Addition of MG132 to the infected samples from 8–12 h p.i., was able to rescue HIF-1α to levels similar to those seen in uninfected cells (Fig. 3A); suggesting that MRV infection induces proteasome-mediated degradation of HIF-1α in hypoxic prostate tumor cells at early times p.i.

Surprisingly, the addition of MG132 at later times in infection was unable to rescue HIF-1α, suggesting that, in addition to proteasome-mediated degradation, a second mode of inhibition of HIF-1α protein accumulation was occurring in MRV infected cells. Because there was no difference in HIF-1α mRNA levels in uninfected and infected cells at this time p.i., (Fig. 2D), we hypothesized that MRV may inhibit HIF-1α mRNA translation. To examine this, we directly measured the levels of HIF-1α protein translated at early and late times p.i. Mock or MRV infected DU 145 cells were incubated in normoxic and hypoxic conditions, and from 6–12 and 18–24 h p.i., the methionine analog, L-AHA was added to the cells to label proteins being translated during the incubation period. MG132 was also added to some samples to prevent proteasome-mediated degradation of the protein that was translated during the labeling time frame. At 12 and 24 h respectively, cells were harvested and L-AHA labeled proteins were conjugated with biotin, and precipitated with streptavidin-conjugated beads, followed by HIF-1α immunoblot (Fig. 3B). In agreement with Fig. 3A, MG132 treatment rescued the HIF-1α synthesized during the 6–12 h p.i. labeling period, confirming that MRV infection induced proteasome-mediated degradation of HIF-1α in hypoxic cells during this time. However, MG132 treatment had little impact on HIF-1α accumulation during the 18–24 h p.i. labeling time, confirming that HIF-1α was not being synthesized during this period, and that at later times during MRV infection, HIF-1α protein translation is inhibited. Taken together, these data indicate that HIF-1α down-regulation in MRV infected hypoxic prostate tumor cells occurs via both proteasome-mediated degradation and inhibition of HIF-1α mRNA translation.

HIF-1α is targeted to the proteasome through both ubiquitin-dependent and -independent mechanisms [37]. To investigate whether MRV induced degradation of HIF-1α occurs through a ubiquitin-dependent or -independent pathway, we utilized a deubiquitinating enzyme inhibitor [NSC632839 hydrochloride (F6)] [38], which prevents removal of ubiquitin chains from polyubiquitinated proteins, inhibiting ubiquitin-dependent, proteasome-mediated degradation. DU145 and PC-3 cells were mock or MRV infected and treated or not with F6 from 8–12 h p.i., at which point proteins were harvested, and HIF-1α levels were analyzed by immunoblot analysis. The addition of F6 rescued HIF-1α from MRV-induced degradation (Fig. 3C) implicating a ubiquitin-dependent pathway in MRV-induced HIF-1α proteasome-mediated degradation.

### The PAS domains of HIF-1α are required for MRV-induced degradation

**Figure 4:**
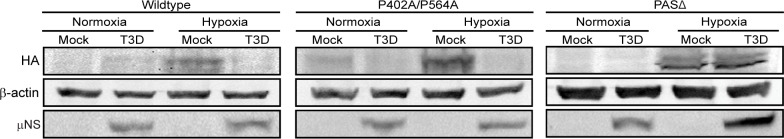
PAS domain region of HIF-1α is required for its MRV-induced down-regulation PC-3 cells were transfected with pHA-HIF-1α, pHA-HIF-1α P402A/P564A or pHA-HIF-1α APAS and incubated under normoxic or hypoxic conditions. At 24 h post-transfection cells were mock infected or infected with MRV T3D and further incubated under normoxic or hypoxic conditions. At 12 h p.i., cells were harvested and proteins were separated on SDS-PAGE and transferred to nitrocellulose. Blots were immunostained with mouse a-HA antibodies, rabbit α-β-actin or rabbit a-μNS antiserum followed by AP-conjugated goat α-rabbit or mouse secondary antibodies.

Several regions within HIF-1α have been identified that are involved in proteasome-mediated degradation. Two of these regions (aa 380–417 and aa 556–572) contain prolines that when hydroxylated by PHD proteins results in recognition by the VHL E3 ubiquitin ligase and targeting of HIF-1α to the proteasome. Another region includes the PAS A and B domains (aa 85–158 and aa 228–298), which are competitively bound by either HSP90 or RACK1 proteins, which promote stability or degradation of HIF-1α, respectively. To determine if the MRV induced degradation of HIF-1α could be mapped to either of these regions within the HIF-1α protein, we utilized HA-tagged HIF-1α encoding plasmids that expressed wild-type HIF-1α (pHA-HIF-1α), PHD hydroxylation mutant HIF-1α (pHA-HIF-1α P402A/P564A) or a PAS A and B domain deletion mutant HIF-1α (pHA-HIF-1α APAS). PC-3 cells were transfected with wildtype or mutant plasmids, and at 24 h post-transfection, cells were mock or MRV infected, and incubated under normoxic and hypoxic conditions. At 12 h p.i., cells were harvested, and accumulation of wild-type and mutant HA-HIF-1α proteins was determined by immunoblotting. In these experiments, MRV infection caused the downregulation of both wild-type HA-HIF-1α and mutant HA-HIF-1α P402A/P564A relative to mock infected cells. However the HA-HIF-1α APAS protein accumulated to similar levels in both mock and MRV infected cells (Fig. 4), suggesting that the PAS domains of HIF-1α are necessary for MRV infection induced degradation of HIF-1α.

### siRNA knockdown of RACK1 prevents MRV-induced degradation of HIF-1α

**Figure 5:**
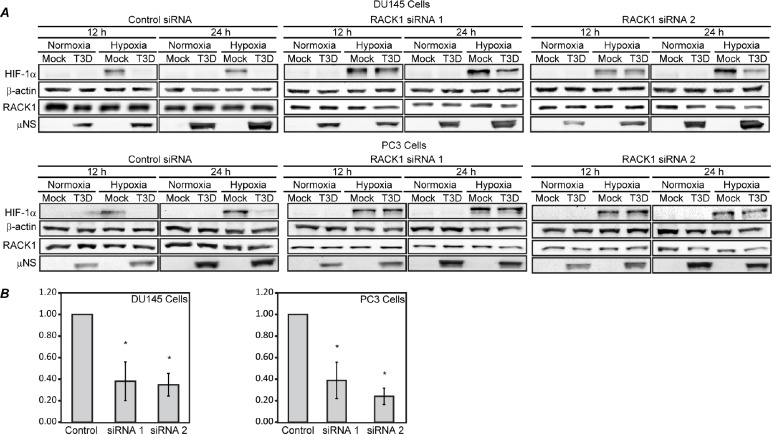
RACK1 knockdown by siRNAs prevents MRV-induced HIF-1α degradation in hypoxic tumor cells DU145 and PC-3 cells were transfected with control or RACK1-specific siRNAs and incubated under normoxic or hypoxic conditions. At 20 h post-transfection, cells were mock infected or infected with MRV T3D and further incubated under normoxic or hypoxic conditions. At 12 h and 24 h p.i., cells were harvested and proteins were separated on SDS-PAGE and transferred to nitrocellulose. A) Blots were immunostained with rabbit a-μNS antiserum, mouse a-HIF-1α antibodies, mouse μ-RACK1 monoclonal antibodies or rabbit a-β-actin antibodies followed by AP-conjugated goat a-rabbit or mouse secondary antibodies. B) RACK1 knockdown by siRNAl and siRNA2 was quantified by measuring the mean of all knockdown samples in each cell type relative to control samples and plotted on a graph. Error bars represent the standard deviation and statistically significant differences (p<0.05) are marked with ‘*’.

Since MRV infection induces degradation of HIF-1α under hypoxic conditions, and additionally induces the degradation of the VHL binding mutant HA-HIF-1α (P402A/P564A), it is unlikely that virus-induced degradation of HIF-1α occurs via the VHL-dependent pathway. However, deletion of the PAS domains of HIF-1α, which are necessary for RACK1 binding, prevents MRV-induced HIF-1α degradation (Fig. 4), suggesting that MRV induced degradation of HIF-1α may occur through a RACK1 dependent pathway. To examine this possibility, we utilized two siRNAs (siRNA 1 and siRNA 2) specific for different regions of the RACK1 mRNA to knockdown RACK1 expression in mock and MRV infected normoxic and hypoxic tumor cells. Quantification of RACK1 levels following knockdown confirmed that cells transfected with RACK1 siRNA 1 or 2 contained on average 60% less RACK1 protein relative to control siRNA treated cells (Fig. 5B). As expected, MRV induced the downregulation of HIF-1α in control siRNA treated samples. In cells transfected with RACK1 siRNAs, at 12 h p.i., where we previously measured the greatest MRV impact on HIF-1α proteasome-mediated degradation, accumulation of HIF-1α was nearly completely rescued (Fig. 5A). At 24 h p.i., there was also a substantial rescue of HIF-1α in the MRV-infected RACK1 knockdown cells relative to control siRNA knockdown cells, however, complete rescue was not observed, likely as a result of the previously identified MRV-induced translational inhibition of HIF-1α mRNA that is occurring by this time p.i. These results indicate that when RACK1 expression is diminished, MRV is unable to induce HIF-1α downregulation. Taken together with data in Fig. 3–4, this strongly suggests that MRV infection induces ubiquitin-dependent, proteasome-mediated degradation of HIF-1α via a RACK1-dependent pathway in hypoxic prostate tumor cells.

### MRV infection induces apoptotic cell death in hypoxic prostate tumor cells

**Figure 6:**
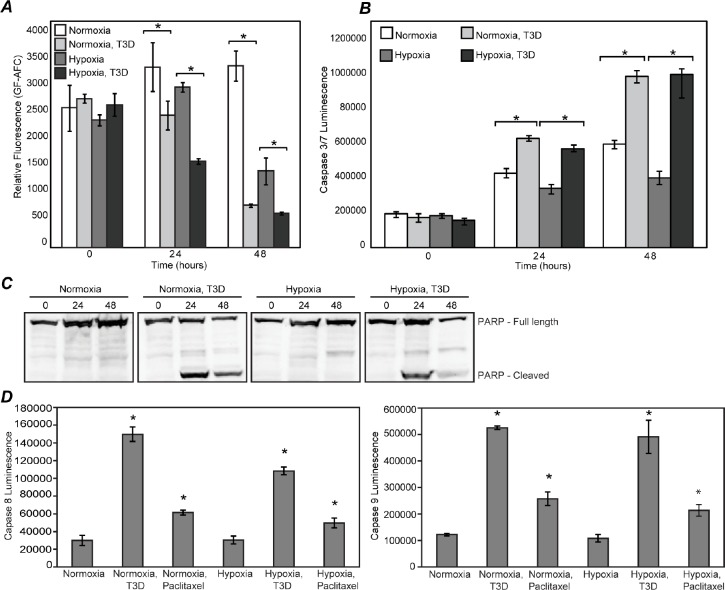
MRV kills hypoxic prostate tumor cells via apoptosis Normoxic or hypoxic DU145 cells were mock-infected or infected with MRV T3D and further incubated in normoxic or hypoxic conditions. A) At 0, 24 and 48 h p.i., cells were subjected to viability assay. B) At 0, 24, and 48 hours, cells were lysed and caspase 3/7 activity was measured. Error bars represent SD of three experimental replicates. Statistically significant differences (p<0.05) are marked with ‘*’. C) At 0, 24, and 48 hours, cells were lysed, and immunoblotted with antibodies against PARP D) At 24 h p.i., cells were lysed and caspase 8 or caspase 9 activities was measured. Error bars represent SD of three experimental replicates. Statistically significant differences (p<0.05) are marked with ‘*’.

Hypoxic cancer cells evade apoptosis by upregulating anti-apoptotic factors [39–41] and downregulating pro-apoptotic factors [42], while MRV infection induces apoptosis [43]. To determine if MRV infection surmounts the hypoxic anti-apoptosis response in PCa cells, we performed a number of experiments. Viability assays based on live-cell protease activity showed that MRV infection caused significantly reduced viability of both hypoxic and normoxic tumor cells relative to uninfected cells (Fig. 6A). Concurrent apoptosis assays showed increased caspase 3/7 activity in MRV infected hypoxic and normoxic tumor cells when compared to uninfected cells (Fig. 6B), suggesting MRV was killing hypoxic tumor cells via an apoptotic pathway. Additional examination of uninfected and infected cell lysates showed cleaved PARP, a hallmark of apoptosis, in MRV infected hypoxic and normoxic tumor cells but not in uninfected cells (Fig. 6C).

Apoptosis in cells occurs via intrinsic and extrinsic pathways. The intrinsic pathway involves the activation of caspase 9 and the extrinsic pathway leads to caspase 8 activation. MRV has been shown to induce apoptosis in normoxic cells in a tissue specific manner and has been shown to be both caspase 8 and 9 dependent [44]. In order to further illuminate the mechanism of MRV induced apoptosis in hypoxic prostate tumor cells, we determined the activation status of caspase 8 and caspase 9 in MRV-infected hypoxic DU145 cells. Paclitaxel, which induces apoptosis via activation of both caspase 8 and 9 was used as a positive control. Caspase activity was measured by Caspase 8/9 Glo assays at 24 and 48 h p.i. From these experiments, it was clear that MRV infection induced the activation of both caspases in normoxic and hypoxic prostate tumor cells (Fig. 6D). Taken together, these data show that MRV infection has the capacity to override the anti-apoptotic effects of hypoxia and induce cell death by activating both the intrinsic and extrinsic apoptosis pathways, ultimately resulting in death of hypoxic prostate tumor cells.

## DISCUSSION

Low oxygen in prostate tumors is strongly predictive of relapse after therapy [45], illustrating the need to identify novel treatment strategies that target hypoxic tumor cells, and the HIF-1α protein that modulates the cellular hypoxic response. In this study, we have identified MRV as one such potential therapy by demonstrating that MRV replicates to high levels in prostate tumor cells grown in hypoxic environments (Fig. 1), induces massive downregulation of the HIF-1α protein via degradation and translational inhibition (Figs. 2, 3), and leads to apoptosis of the cells (Fig. 6). It is important to note that our data does not suggest that MRV specifically targets hypoxic cells, merely that the ability of MRV to replicate and induce apoptosis of tumor cells can be expanded to include those growing in a hypoxic microenvironment. These findings illustrate that the cellular adaptations that occur during hypoxia are not detrimental to successful MRV replication. This natural ability to replicate in hypoxic cells is similar to that seen in vesicular stomatitis virus (VSV), which has previously been shown to replicate in hypoxic HeLa cells and in hypoxic regions of C6 gliobastoma tumor xenografts [46]. Like VSV, the natural ability of MRV to replicate in hypoxic tumor cells circumvents the need to alter the virus for specific replication in the hypoxic microenvironment, as is being investigated in other promising oncolytic viruses [46–48].

The downregulation of HIF-1α during MRV infection of PCa cells is mediated by proteasome-mediated degradation, and translational inhibition (Fig. 3). It remains unclear if HIF-1α degradation is a specific consequence of MRV infection, or occurs as a non-specific result of MRV impact on other proteins involved in HIF-1α regulation. One particularly attractive candidate HIF-la regulatory protein is HSP90, which has been shown to be involved in folding of the MRV trimeric attachment protein σ1 [49]. Usurpation of HSP90 in infected cells for σ1 folding may indirectly lead to RACK 1-mediated degradation of HIF-1α. Inhibition of HIF-1α translation at later times in MRV infection may occur as a result of modification of the translational machinery during virus infection. MRV has been shown to induce translational shutoff in many cell types, although we have been unable to detect a similar general inhibition of translation by MRV in the prostate tumor cells used in these studies (Gupta-Saraf and Miller, unpublished). It is possible that sequestration of translation initiation factors by viral mRNA results in downregulation of HIF-1α translation. Further elucidation of how MRV infection modifies the cellular translation machinery should shed light on how HIF-1α mRNA translation is inhibited in infected cells.

The PAS domain of HIF-1α, which is required for RACK1 and HSP90 binding [50], is required for MRV-induced degradation (Fig. 4). Additionally, siRNA knockdown of RACK 1 inhibits MRV-induced HIF-1α degradation (Fig. 5). This suggests that MRV infection may result in modification of the interaction between RACK1 or HSP90 and HIF-1α. MRV infection may increase the RACK 1/HIF-1α interaction or interfere with the HSP90/HIF-1α interaction, leading to increased HIF-1α degradation. Moreover, post-translational modifications of both HSP90 (acetylation) and RACK 1 (phosphorylation) play important roles in regulation of HIF-1α association [51, 52] and it is possible that MRV infection alters these modifications. Finally, cellular modulators of interactions between HIF-1α, RACK 1 and HSP90 that regulate HIF-1α protein levels may also be altered by MRV infection. Potential examples of this are the mammalian septin family member, SEPT9_v1, which has been reported to bind HIF-1α and prevent RACK 1 mediated proteasome targeting [53] or the SSAT2 protein, which binds to HIF-1α and RACK1 and promotes HIF-1α ubiquitination [54]. Elucidating these possibilities will be essential for gaining a full understanding of how MRV induces HIF-1α degradation and is currently under investigation.

Hypoxia selects for cancer cells that can evade apoptosis [55]. We show that MRV can extend its oncolytic properties to hypoxic PCa cells via induction of apoptosis by activating both caspase 8 and 9 (Fig. 6). This suggests that MRV infection can override the anti-apoptotic pathways induced by hypoxia. Because HIF-1α plays a role in inhibition of apoptosis in hypoxic cells, it is possible that MRV-induced downregulation of HIF-1α allows cells to activate their normal apoptosis response to hypoxia in infected cells. The exact pathway of MRV-induced apoptosis in hypoxic prostate tumor cells is currently under investigation.

We show that MRV downregulates the attractive cancer therapeutic target HIF-1α and induces apoptosis in hypoxic prostate tumor cells. These findings augment existing information regarding the capacity of MRV to infect different tumor cell types growing in diverse physiologically relevant microenvironments. Validation of MRV oncolytic therapy of PCa in clinical trials is ongoing and has been shown to have a positive effect on disease regression [29]. MRV has also been shown to work in synergism with chemotherapy drugs such as docetaxel, which is the first line chemotherapy for treatment of androgen resistant PCa [30]. Our study extends the potential value of MRV treatment in PCa to include hypoxic prostate tumor cells, and provides a rationale to pursue future *in vivo* animal studies investigating the efficacy of MRV against hypoxic microregions within PCa tumors either alone or in combination with other standard chemotherapy treatments that are not effective against hypoxic cells. As hypoxia is present throughout the course of PCa, our data further suggests that MRV therapy may also be a strong candidate for targeting hypoxic cells and HIF-1α in PCa clinical trials.

## MATERIALS AND METHODS

### Cells and reagents

DU 145 cells were maintained in Eagle's modified essential medium (Invitrogen), PC3 cells were maintained in F-12K media, and LNCaP cells were maintained in RPMI media (ATCC), containing 10% fetal bovine serum (Atlanta Biologicals) and penicillin-streptomycin (100 IU/ml, Mediatech). L929 cells were maintained in Joklik's minimal essential medium (Irvine Scientific) containing 2% bovine calf serum, 2% fetal bovine serum (Atlanta Biologicals), 2 mM L-Glutamine (Mediatech) and penicillin-streptomycin (100 IU/ml, Mediatech). All cell lines were obtained from the American Type Culture Collection. Primary antibodies used were as follows: mouse monoclonal anti-RACK 1 (BD Biosciences), mouse monoclonal anti-HIF-1α (Becton, Dickinson and Company Biosciences), rabbit polyclonal anti-β-actin (Cell Signaling Technologies), rabbit monoclonal anti-PARP (Cell Signaling Technologies), rabbit polyclonal anti-μNS [56], and mouse anti-HA (Cell Signaling Technologies). Secondary antibodies used for immunoblot experiments were alkaline phosphatase (AP)-conjugated goat anti-mouse or anti-rabbit IgG (Bio-Rad). Secondary antibodies used in immunofluorescence experiments were Alexa 488-conjugated donkey anti-mouse, and Alexa 594-conjugated donkey anti-rabbit immunoglobulin G (IgG) antibodies (Invitrogen). Proteasome inhibitor, MG132 (Enzo Life Sciences), was used at a final concentration of 10 μM, cobalt chloride (CoCl_2_) at a final concentration of 500 μM and NSC 632839 hydrochloride (F6) (BostonBiochem) at a final concentration of 30 μM.

### Infection and Transfection

MRV virions (T3D strain) are from our laboratory stocks. Purified virions were prepared as described [57], using Vertrel reagent (DuPont) in place of Freon, and stored in dialysis buffer (150 mM NaCl; 10 mM Tris pH 7.4; 10 mM MgCl_2_) at 4°C. Cells were seeded onto 60-mm, 35-mm, or 9.6-cm^2^ cell culture dishes 24 h before infection. Cells were infected with MRV virions at a cell infectious unit (CIU) of 1 based on titers determined on cell lines used as previously described [58].

### Hypoxia

Hypoxic conditions were obtained by incubating cells in 1% O_2_ and 5% CO_2_ at 37°C in a Galaxy 48R CO_2_ Incubator (New Brunswick Scientific) equipped with 1–19% O_2_ controls. For all experiments the cells were adapted to hypoxia for 4 h prior to infection.

### Immuno blotting

Cells were lysed in 100 μL 2X SDS protein loading buffer (125 mM Tris.HCl [pH 6.8], 200 mM DTT, 4% SDS, 0.2% Bromophenol blue, 20% Glycerol). Immunoblots were performed as previously described [58]. Blots were exposed to Lumi-Phos™WB Chemiluminescent Substrate (Thermo Scientific), and images were collected and quantified using a ChemiDoc XRS camera and Quantity One software (Bio-Rad). All experiments were independently performed at least 3 times and representative results are shown.

### Immunofluorescence

Cells were fixed and processed for immunofluorescence as previously described [56]. Samples were imaged with a Zeiss Axiovert 200 inverted microscope equipped with fluorescence optics. Images were prepared using Photoshop and Illustrator software (Adobe Systems). All experiments were independently performed at least 3 times and representative results are shown.

### Virus Replication Assay

Samples were harvested and subjected to 3 freeze thaw cycles. Serial ten-fold dilutions in phosphate-buffered saline (PBS) (137 mM NaCl, 3 mM KCl, 8 mM Na_2_HPO_4_ [pH 7.5]) containing 2 mM MgCl_2_ were made and plaque forming units (PFU) were determined by standard plaque assay on L929 cells [59]. The experiment was done thrice independently and the average of the three experiments was plotted on a bar graph with error bars depicting the standard error of the averages.

### Quantitative Real-Time PCR

RNA was harvested using Trizol (Invitrogen) as per manufacturer's instruction. 1 μg RNA was treated with DNAase I (New England Biolabs) then used to make cDNA with Superscript III Reverse Transcriptase (Invitrogen) as per manufacture's protocol. Primers used were as follows: β-actin (409 bp), the forward primer was ACCAACTGGGACGACATGGAGAAA and the reverse primer was TTAATGTCACGCACGATTTCCCGC; HIF-1α (564 bp), the forward primer was GAACCTGATGCTTTAACT and the reverse primer was CAACTGATCGAAGGAACG. The cDNA was amplified by qPCR using 0.1 μM primers, 0.7X SYBR green, 200 uM dNTP, IX GoTaq Reaction Buffer, 1.25 U GoTAQ polymerase (Promega) and 2 μL of reverse transcription reaction mixture as template in an Opticon cycler (Biorad). The C(t) value of HIF-1α was averaged using the C(t) value of β-actin and plotted on a graph. The experiment was done thrice independently and each experiment included 2 replicates. The average of the three experiments was plotted on a bar graph with error bars depicting the standard error of the averages.

### Transfection

Commercially available control and RACK1 specific siRNAs (Cat. No. 4392421-s20342, s20341, Ambion) were complexed with Lipofectamine 2000 (Invitrogen) according to the manufacturer's protocol and added to cell suspension prepared by trypsinization in 1 mL of Opti-MEM. After 4 hours incubation, 1 mL of Eagle's MEM with 20%) FBS and no antibiotics was added. After 24 h incubation at 37°C, new siRNA:Lipofectamine 2000 complexes were prepared and added to the transfected adherent cells. Following a 24 h incubation in normoxic conditions, transfected cells were mock-infected or infected with MRV and incubated in either normoxic or hypoxic conditions for 12 or 24 h, at which point cells were harvested and lysed in protein loading dye and subjected to immunoblot analysis. Blots were exposed to Lumi-Phos™WB Chemiluminescent Substrate (Thermo Scientific), and images were collected and quantified using a ChemiDoc XRS camera and QuantityOne software (Bio-Rad). The experiment was repeated independently 3 times and representative results are shown. Plasmid transfection was performed using TransIT-LT1 (Mirus) as per manufacturer's protocol.

### Viability and caspase activity assay

Cells were subjected to the ApoTox-Glo Triplex Assay (Promega) or Caspase 8/9 Glo Assay (Promega) according to the manufacturer's protocol. Fluorescence and luminescence was recorded using GloMax Multi+microplate reader (Promega). Graphs were constructed using Microsoft Excel. Results shown are the average and standard deviation of three experimental replicates. Statistical significance was calculated using averages of 3 experimental replicates.

### Plasmid Construction

A firefly luciferase reporter plasmid (pGL4.26 luc2/minP/Hygro) was purchased from Promega. Upper and lower oligonucleotides were designed which contain three copies of the hypoxia response element [60], 5′ GTACGTGCT 3′, flanked on each end by NheI and HindIII restriction sites (New England Biolabs). Oligonucleotides were annealed and ligated into NheI and Hindlll digested pGL4.26 luc2/minP/Hygro to create pHRE-Luc. pHA-HIF-1α [Addgene plasmid 18949, [61]] and pHA-HIF-1α P402A/P564A [Addgene plasmid 18955, [62]] were obtained from Dr. William G Kaelin, Addgene. pHA-HIF-1α APAS was created by Hindlll digestion of pHA-HIF-1α to remove aa 85–298 containing the PAS domain. A synthetic double-stranded DNA flanked on the end with BamH1 and Agel digestion sites and containing HIF-1α nt 1-1096, with the PAS domain (nt 253–894) deleted was purchased (gBlock, Integrated DNA Technologies), digested with BamHI and AgeI, and ligated into the digested pHA-HIF-1α. Following ligation, transformation, and screening, all plasmids were verified by sequencing.

### Luciferase Assay

Cells were transfected with pHRE-Luc using Lipofectamine 2000 (Invitrogen) according to the supplier's protocol. Luciferase expression was measured using the One-Glo Luciferase Assay Kit and the luminescence function of a GloMax Multi+ microplate reader (Promega). Following recording of luminescence, cells were lysed and total protein measured using Bradford Assay (Biorad) and the absorbance function of a GloMax Multi+ microplate reader. Luminescence levels were normalized to total protein and plotted on a graph. Results shown are means and standard deviation of three experimental replicates. The statistical significance was calculated using the average of 3 experimental replicates.

### L-Azidohomoalanine (L-AHA) protein labeling and precipitation

50 μM L-AHA (Invitrogen) was added to cells at 6 or 18 h p.i. then cells were harvested in lysis buffer (1% SDS in 50 mM Tris-HC1, pH 8.0) at 12 h or 24 h p.i., respectively. L-AHA-1αbeled proteins were conjugated with biotin as per manufacture's protocol. Following conjugation, proteins were precipitated and resuspended in 100 uL lysis buffer, diluted to 800 (μl in Tris-buffered saline (20 mM Tris, 137 mM NaCl [pH 7.6]) containing 0.1% Tween (TBS-T) and incubated for 2–3 h at room temperature with streptavidin magnetic beads (Pierce) prepared as per manufacturer's instruction. Beads were collected and washed 6 times with TBS-T, then suspended in 2X SDS protein loading buffer for immunoblot analysis.

### Statistical Analysis

Statistical significance was determined using student's t-test and two-tailed p value calculated with GraphPad software. Differences in groups for which p < 0.05 were considered to be statistically significant and are indicated with an asterisk in the figures.
